# A program to identify prognostic and predictive gene signatures

**DOI:** 10.1186/1756-0500-7-546

**Published:** 2014-08-18

**Authors:** Sam D Chorlton, Robin M Hallett, John A Hassell

**Affiliations:** Department of Biochemistry and Biomedical Sciences, Centre for Functional Genomics, McMaster University, 1200 Main Street West, Hamilton, L8N 3Z5 Ontario Canada

## Abstract

**Background:**

The advent of high-throughput technologies to profile human tumors has generated unprecedented insight into our molecular understanding of cancer. However, analysis of such high dimensional data is challenging and requires significant expertise which is not routinely available to many cancer researchers.

**Results:**

To overcome this limitation, we developed a freely accessible and user friendly Program to Identify Molecular Signatures (PIMS). Importantly, such signatures allow important insight into cancer biology, as well as provide clinical tools to identify potential biomarkers that might provide means to accurately stratify patients into different risk or treatment groups. We evaluated the performance of PIMS by identifying and testing predictive and prognostic gene signatures for breast cancer, using multiple breast tumor microarray cohorts representing hundreds of patients. Importantly, PIMS identified signatures classified patients into high and low risk groups with at least similar performance to other commonly used gene signature selection techniques.

**Conclusions:**

Our program is contained entirely within a Microsoft Excel file and therefore requires no installation of any additional programs or training. Hence, PIMS provides an accessible tool for cancer researchers to identify predictive and prognostic gene signatures to advance their research.

**Electronic supplementary material:**

The online version of this article (doi:10.1186/1756-0500-7-546) contains supplementary material, which is available to authorized users.

## Background

Cancer oncologists are faced with the challenging task of predicting which patients are most likely to benefit from various treatment modalities, as well as avoid overtreating patients who are unlikely to benefit from aggressive therapy. For example, in breast cancer the traditional parameters used by pathologists to determine patient prognosis include age, tumor size, as well as various histopathological measurements such as clinical grade and hormone receptor status [1, 2]. More recently, the development of gene expression profiling technologies such as microarrays and quantitative RT-PCR have led to the use of molecular signatures as additional means for providing prognostic information for breast cancer patients [3–15]. Indeed, multigene predictors, which are also commonly called gene signatures, are already being used clinically in some instances, such as the MammaPrint® and OncotypeDX™ tests. Apart from breast cancer, gene signatures have also been applied to other cancer types to determine patient prognosis and other clinical parameters of interest [16, 17]. Additionally, examination of transcripts that comprise gene signatures can reveal biological processes which underlie clinical phenomena, and potentially uncover new therapeutic avenues. Hence, gene signatures provide an important tool to advance clinical as well as basic cancer research. However, identifying predictive or prognostic gene signatures requires the use of specialized software and bioinformatics training, which ultimately hampers their adoption where such infrastructure or skills are lacking.

We hypothesized that an Excel program, which identified predictive and prognostic gene signatures and did not require the installation or use of any other software packages, would increase the accessibility of this type of research. To this end, we adapted and improved an algorithm we previously published [12] into a freely accessible and user-friendly Excel program: Program to Identify Molecular Signatures (PIMS). Here, we demonstrate its use to identify prognostic gene signatures, which stratify breast cancer patients into high and low risk groups, as well as predictive gene signatures, which stratify breast cancer patients into chemotherapy responsive and non-responsive groups. These findings suggest that our program is robust and can be used to develop predictive and prognostic gene signatures for user defined contexts. Hence, we conclude that PIMS provides an accessible tool for cancer researchers to identify predictive and prognostic gene signatures to advance their research aims.

## Methods

### Microarray and clinical data

All data was obtained de-identified and obtained from publically available sources through the gene expression omnibus. We downloaded the following datasets as well as associated clinical data from the gene expression omnibus (GSE2034 [n = 286] [11], GSE7390 [n = 198] [18], GSE25055 [n = 310] [19], GSE25065 [n = 1] [19]), and GSE14333(n = 290). All datasets were normalized using RMA [20] using the public gene pattern server (http://genepattern.broadinstitute.org).

### Prognostic signatures

GSE7390 (n = 198) was used for training and GSE2034 (n = 286) was used for testing. Both GSE2034 and GSE7390 comprise patients with node negative disease who had received no treatment with endocrine agents or chemotherapy. When possible we used distant metastasis free survival at 10 years as the clinical endpoint for this study. A summary of the clinical characteristics of these cohorts are provide in Table 1. For the colon study, GSE14333 was randomly divided into two equal sized cohorts. One cohort was used as a training set, whereas the other was used as a validation set.

**Table 1:** **Summary of training and validation cohorts used for prognostic signature**

Characteristic	Validation cohort	Training cohort
	GSE2034	GSE7390
Samples	286	198
Array type	U133A	U133A
ER positive	209 (73%)	134 (68%)
Median survival	86 months	144 months
Survival at 10 yrs	62%	69%
	**Total arrays: 484**	

### Predictive signatures

GSE25055 was used as the training dataset and GSE25065 was used as the validation dataset. Both datasets comprise patients treated with neoadjuvant chemotherapy comprising an anthracycline and taxane. Patients with a post chemotherapy residual cancer burden (RCB) of 0 or I were considered to be responders, whereas those with an RCB of II or III were considered to be non-responders. A summary of the clinical characteristics of these cohorts are provide in Table 2.

**Table 2:** **Summary of training and validation cohorts used for predictive signature**

Characteristic	Discovery cohort	Validation cohort
	GSE25055	GSE25065
Samples	310	198
Array type	U133A	U133A
ER positive	174 (56%)	123 (62%)
RCB0/I vs RCBII/III	86 (29%)	32 (27%)
	**Total arrays: 508**	

### Feature selection algorithm

We significantly improved a previously published feature selection algorithm [12] by adding leave-one-out cross-validation as well as improved means of calculating signature scores, to produce software capable of identifying prognostic/predictive gene signatures. Initially, gene expression for all patients is standardized across each probe set, such that the mean and standard deviation of each probe set is set to 0 and 1 respectively. Gene expression is then binned into the categories high, typical, and low based on the 95% confidence interval of expression for a given gene. For example, high gene expression indicates that the expression of a gene exceeds the 95% confidence interval of expression for that gene among all patients, and low expression indicates that the expression of a gene was less than the 95% confidence interval of that gene among all patients. Genes with expression within the 95% confidence interval of expression were considered to have typical expression. A predictive score (initially set at 0) for each probe set/gene is then calculated in the following way (Additional file 1: Figure S1):

1. Patients who had the event and have high expression of a gene increase the predictive score of that gene by 1.
2. Patients who had the event and have low expression of a gene decrease the predictive score of that gene by 1
3. Patients who did not have the event and have high expression of a gene decrease the predictive score of that gene by 1.
4. Patients who did not have the event and have low expression of a gene increase the predictive score of that gene by 1.
5. Typical expression of a gene in any patient does not change its predictive score.

In this fashion, high absolute predictive gene scores may be achieved by either high or low expression of a given gene being related to patient outcome. Finally, we rank the genes by predictive score and select the most predictive genes. The magnitude of the difference in mean gene expression between the high and low risk groups is used as a tie-breaker. In this fashion, the expression of probe sets that receive the highest scores are associated with high risk tumors (those that reccur within 10 years), and the expression of probe sets that receive the lowest scores are associated with low risk tumors (those that do not reccur within 10 years). In order to estimate the performance of a given signature in an unbiased fashion, and reduce over-fitting, we added capacity for PIMS to perform leave one-out-cross validation. Screenshots of this process as well as detailed instructions can be found in Additional file 2 (PIMS user guide).

To assign signature scores to patients, the expression values for each gene were transformed such that the mean and standard deviation were set to 0 and 1 in each dataset, respectively. A signature score was calculated for each patient as follows:


Where *x* is the transformed expression, *n* is the number of probe sets, *P* is the set of probes with reported positive correlation to the target probe set, and *N* is the set of probes with reported negative correlation to the target probe set [13, 15].

### Software

The program is contained entirely within an Excel file, therefore requiring no installation. All that is required to operate our program is Excel 2007 or later. Additionally, our program is freely accessible and is included as a supplementary file, which accompanies this manuscript. The code for our program is written in Visual Basic for Applications and is easily accessible from within Excel.

#### Prediction Analysis of Microarrays (PAM)

PAM was installed and used in R according to the available manual [21].

#### Binary Regression (BR)

The binary regression software was a generous gift from the West lab (http://www.stat.duke.edu/~mw/), and was used as a MATLAB plug-in [22].

### Performance assessment

For the prognostic validation, we calculated the hazard ratio (HR), logrank p-value (median cut-point), area under the ROC curve (AUC), and specificity at 80% sensitivity, to determine the significance of the difference in survival between predicted good and poor survival groups. For the predictive validation, we calculated the odds ratio (OR) and Fisher’s exact test to assess performance. Survival analysis and all associated statistical tests were performed using IBM SPSS Statistics and R.

## Results

### Identification of a prognostic gene signature

**Figure 1:**
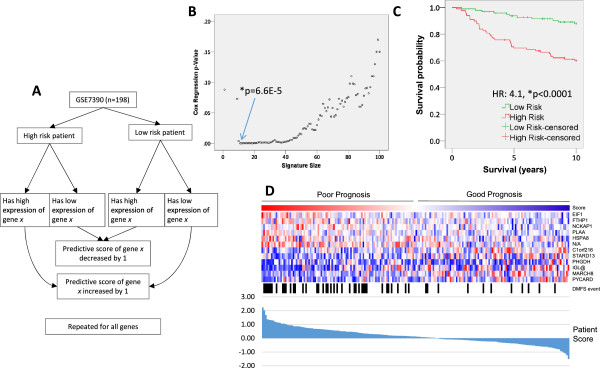
**Identification of prognostic gene signatures using PIMS. A)** Summary of PIMS algorithm. **B)** Cox regression p-values compared to n-length signatures selected by PIMS (n = 12 genes, p < 0.0001). **C)** Survival analysis of patients stratified into high and low risk groups by the PIMS 12-gene signature (HR:4.1, p = 0.000007). **D)** Heatmap and individual patient signature scores for 12-gene signature.

We sought to develop user-friendly and accessible software that could reliably identify predictive and prognostic gene signatures, which we tested by identifying and testing predictive and prognostic gene signatures from global gene expression profiles of breast tumors. In short, PIMS computes a predictive score for each gene, such that genes which receive the highest absolute predictive scores are tightly linked to poor or good outcome. Based on the predictive scores, it is possible to select an n-feature gene signature, which comprises n-genes that received predictive scores of the greatest magnitude. The expression of genes comprising a signature can then be evaluated in a given patient, resulting in that patient receiving a signature score. Gene signatures can then be validated by testing whether or not the signatures scores are associated with the clinical feature of interest. In this case, we used patients who were or were not distant metastasis free within 10 years after diagnosis as phenotypic classes, and used our software to identify prognostic genes that stratify patients into these classes (Figure 1A, Additional file 1: Figure S1). Because it is difficult to know, *a priori*, the optimal number of n-features (in this case features are Affymetrix probesets/genes) to include in a gene signature, we introduced leave-one-out cross-validation (LOOCV) as a means to identify an optimal number of features to include. To this end, PIMS produced scores that predicted the outcome of each patient using LOOCV. To determine the optimal number of genes to include in our signature, we identified 50 signatures of length n = 1,2,3,…,50 and compared the p-values from Cox regression of the survival on predicted scores for each patient for each n-length signature (Figure 1B). We found that a signature comprising 12 genes yielded the lowest p-value (p < 0.0001, Figure 1B), suggesting that the expression of 12 genes optimally stratify patients into good and poor prognosis groups. Indeed, we found that a 12 gene signature selected and evaluated by PIMS (LOOCV) could stratify patients into high and low risk groups with highly statistically significant differences in survival (p < 0.00000001, Figure 1C and D). Taken together, these data suggest that PIMS provides a simple and robust means of identifying prognostic gene signatures in cancer patients.

Pathway analysis of the prognostic genes selected by PIMS demonstrated that these genes were enriched in several biological processes previously linked to breast cancer patient outcome (Additional file 3: Table S1). These included regulation of adherens junctions as well as nuclear regulation of SMAD2/3 signaling, which occurs downstream of TGFβ signaling. Given the previous reported linkage between adherens junction, TGFβ signaling and breast patient prognosis, these results confirm the capacity of PIMS to select prognostic genes.

### Comparison with other models

**Figure 2:**
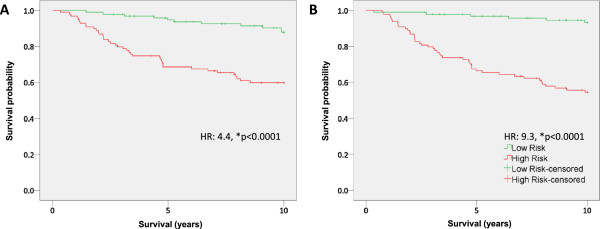
**PAM and binary regression identify prognostic gene signatures. A)** Survival analysis of patients stratified into high and low risk groups by a 12 gene signature identified using binary regression (HR: 4.4, p = 0.00003). **B)** Survival analysis of patients stratified into high and low risk groups by a 12 gene signature identified using PAM (HR: 9.1 p < 0.000001).

To compare the predictive accuracy of PIMS identified signatures with those identified by other commonly used software packages, we compared the PIMS 12-gene signature (described above) with similar 12 gene signatures identified using either binary regression or Predictive Analysis of Microarrays (PAM). In each case, we used GSE7390 as the discovery dataset and GSE2034 as a validation dataset. To compare the various means for signature selection we calculated the hazard ratio (HR) and logrank p-value (median cut-point), area under the ROC curve (AUC) for survival at 10 years, as well as test specificity at 80% sensitivity. We found that both binary regression and PAM identified signatures which were highly prognostic in the discovery dataset (Figure 2A and B, Table 3). Based on our evaluation criteria we found that each feature selection method identified signatures that were significantly associated with outcome (Table 3, GSE7390 row).

**Table 3:** **Comparison of PIMS, PAM, and binary regression identified prognostic signatures**

		GSE7390 (Discovery)	GSE2034 (Validation)
**PIMS**	Hazard ratio	7.6	2
	p-value Log-rank Test	<0.0001	0.0003
	AUC	0.81	0.65
	AUC p-value	<0.0001	<0.0001
	Specificity at 80% sensitivity	0.74	0.37
	Cox regression p-value	<0.0001	0.0002
**PAM**	Hazard ratio	9.3	2
	p-value Log-rank Test	<0.0001	0.0004
	AUC	0.82	0.62
	AUC p-value	<0.0001	0.001
	Specificity at 80% sensitivity	0.69	0.31
	Cox regression p-value	<0.0001	0.0002
**Binary regression**	Hazard ratio	4.4	1.4
	p-value Log-rank Test	<0.0001	0.063
	AUC	0.77	0.57
	AUC p-value	<0.0001	0.04
	Specificity at 80% sensitivity	0.56	0.32
	Cox regression p-value	<0.0001	0.01

**Figure 3:**
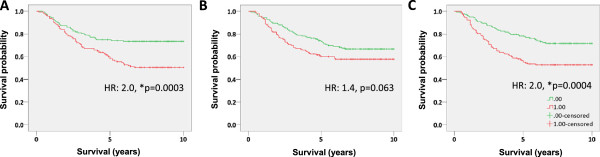
**Survival analysis of PIMS PAM and binary regression identified signatures on a independent cohort. A)** Survival analysis of patients stratified into high and low risk groups by a 12 gene signature identified using PIMS (HR: 2.0, p = 0.0030). **B)** Survival analysis of patients stratified into high and low risk groups by a 12 gene signature identified using binary regression (HR: 1.4, p = 0.063). **C)** Survival analysis of patients stratified into high and low risk groups by a 12 gene signature identified using PAM (HR: 2.0 p = 0.0036).

Because independent validation of a gene signature is a more accurate measurement of its actual prognostic capacity, we sought to evaluate PIMS, binary regression and PAM selected signatures on an independent cohort of breast cancer patients (GSE2034). GS2034 is a cohort of 286 chemotherapy naïve, node-negative breast cancer patients, thus having characteristics similar to those used for the training data set. Analysis of this data revealed that the PIMS selected signature performed nominally better than either the PAM or binary regression selected signatures, albeit marginally so (Table 1: winners bolded). In the validation tests, PIMS, PAM and binary regression selected signatures produced hazard ratios of 2.0 (p = 0.0003), 2.0 (p = 0.0004), and 1.4 (p = 0.06), respectively. AUC analysis, Cox regression, and sensitivity and specificity comparisons all suggested that each feature selection method identified a signature that was also associated with patient outcome in the validation cohort (Figure 3). Notably, the PIMS selected signature was the nominal winner of each category. We also observed that each signature had poorer performance during independent validation, suggesting that each signature suffered from over-fitting during training, a phenomena which is common to signatures identified from high dimensional data. Nonetheless, our PIMS selected signature performed with at least similar accuracy to signatures selected with other commonly used feature selection algorithms.

### Comparison of PIMS with randomly generated signatures

**Figure 4:**
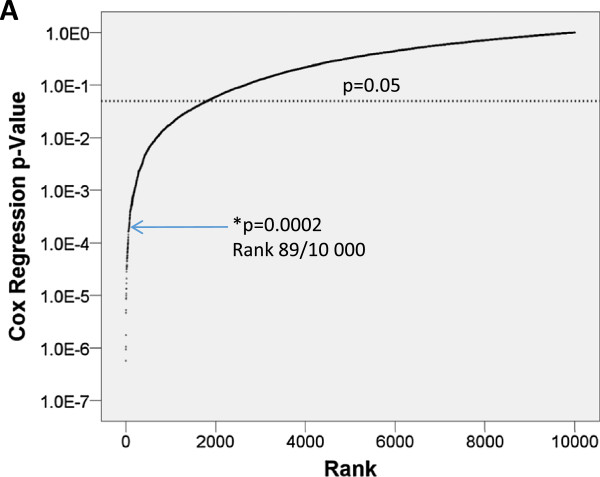
**PIMS signature outperforms randomly generated signatures. A)** Comparison of the PIMS signature with 10,000 randomly generated signatures. Cox-regression p-value for the PIMS signature is shown by blue arrow (p = 0.00020, rank: 89/10,000).

Recently, many groups have found that randomly generated gene signatures significantly correlate with outcome using standard tests, such as the ones we used as described [23, 24]. To confirm that PIMS identified signatures that performed better that those selected by random means, we generated 10,000 random 12 gene signatures and used Cox regression to test their association with patient outcome (GSE2034, validation set). In support of these studies, we found that 20% of the randomly generated signatures were significantly associated with outcome. However, we also found that the PIMS identified signature performance placed it within the 99^th^ percentile of randomly generated signatures (Figure 4A, blue arrow). Hence, we concluded that PIMS selects gene signatures with robust prognostic capacity, and PIMS selected signatures are not simply prognostic by chance.

### Identification of a predictive gene signature

**Figure 5:**
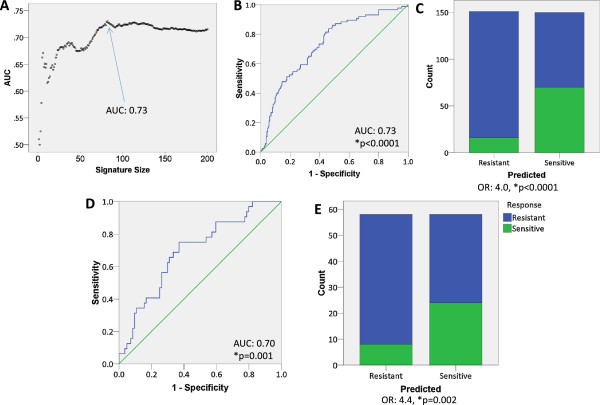
**Identification of a predictive gene signature using PIMS. A)** The optimal signature length was identified by using ROC analysis for each n-length signature (n = 1-200, n = 82). **B)** ROC analysis of 82 gene signature evaluated using LOOCV (AUC: 0.73, p < 0.000001). **C)** LOOCV of the 82 gene signature stratified patients into responder and non-responder groups (OR: 4.0, p < 0.00001). **D)** ROC analysis of 82 gene signature in validation cohort (AUC: 0.70, p = 0.0012). **E)** 82 gene signature stratified validation cohort patients into responder and non-responder groups (OR: 4.4, p = 0.002).

Our previous data suggested that PIMS could be used to identify robust prognostic gene signatures. Because identifying predictive gene signatures is also of significant interest to cancer researchers, we tested whether PIMS could identify gene signatures predictive of therapy response. We used PIMS to identify a gene signature that could predict breast cancer tumor response to pre-operative or neoadjuvant chemotherapy. For this aim, we used GSE25055 (n = 310) and GSE25065 (n = 198) as training and validation cohorts, respectively. Each cohort comprises patients that were treated with neoadjuvant anthracycline and taxane chemotherapy, and were evaluated for residual cancer burden (RCB) post-therapy. In this case, we considered patients with RCB0/I post therapy as responders or chemotherapy sensitive, and those with RCBII/III as non-responders, or chemotherapy resistant [25]. To identify an optimal signature we used PIMS to identify and LOOCV to test n-length signatures (n = 1,2,3,....,200), and measured the AUC using ROC analysis of each n-length signature. We identified 82 probesets as the optimum signature size with an AUC at 0.73 (Figure 5A). Moreover, PIMS LOOCV could stratify patients (median cut-point) into chemotherapy sensitive and chemotherapy resistant patient groups (OR: 4.0, p < 0.0001, Figure 5B, C). To confirm that the PIMS identified signature was robustly predictive, we also tested the capacity of the 82 gene signature to identify responders in the additional validation dataset. The 82 gene signature could accurately segregate the patients from the validation cohort into responders and non-responders (AUC = 0.70, OR: 4.4, *p = 0.002, Figure 5D and E). We also compared the accuracy of the PIMS identified signature to that of similar signatures derived using either PAM or binary regression (Table 4). In this comparison, which included ROC curve analysis and contingency analysis, we found that the PIMS, PAM, and binary regression identified predictive signatures were directly comparable in terms predictive accuracy.

**Table 4:** **Comparison of PIMS, PAM, and binary regression identified predictive signatures**

	Test	GSE25065 (Validation cohort)
**PIMS**	AUC	0.70, p = 0.001
	Odds ratio	4.4
	Fisher’s exact test	p = 0.002
**PAM**	AUC	0.72, p = 0.0002
	Odds ratio	4.4
	Fisher’s exact test	p = 0.002
**Binary regression**	AUC	0.71, p = 0.0004
	Odds ratio	4.4
	Fisher’s exact test	p = 0.002

Hence, these data suggest that PIMS identified signatures have the capacity to identify predictive gene signatures. Taken with our previous data, we conclude that PIMS provides a robust means of identifying predictive and prognostic gene signatures in breast cancer.

### PIMS identifies prognostic gene signatures in additional tumor types

To confirm that the utility of PIMS was not limited to breast cancers, we tested its capacity to identify prognostic signatures for risk stratification of colon cancer patients. Briefly, we obtained publically available gene expression profiling data for which clinical follow-up data was also available (GSE14333). We randomly divided this cohort into equally sized training and validation cohorts, and implemented PIMS to identify a 12 feature signature that robustly stratified training patients into good and poor outcome groups. Application of this 12 gene signature to the validation cohort demonstrated striking stratification of these patients into high and low risk groups (Additional file 4: Figure S2, HR: 1.3, *p = 0.0004, log-rank test). Taken together, these data demonstrate the capacity for PIMS to identify prognostic signatures in colon cancers. Overall, we conclude that PIMS provides a robust and reproducible method to identify prognostic and predictive gene signatures.

## Discussion

Here, we report a freely accessible and user friendly program to identify predictive and prognostic gene signatures. An important characteristic of our program is that it is all contained within a single Microsoft Excel file. Excel is highly used and widely available: therefore the implementation of our program is very straightforward. By contrast, the vast majority of current feature selection techniques require the use of various clustering and classification algorithms that require installation of advanced statistical software packages as well as a significant time investment for training with the same software.

A comparison of PIMS with PAM and binary regression suggested that PIMS identified signatures that performed with comparable accuracy to other commonly used feature selection techniques. It is noteworthy that for each signature, regardless of their method of derivation, the predictive accuracy diminished between the training and validation groups, suggesting that over-fit occurred during training. This is a common property of feature selection algorithms [10, 26]. To confirm that PIMS selected signatures were robustly associated with the defined clinical variables, we also generated 10,000 randomly selected signatures and compared their predictive capacity with the PIMS selected signature. In this case, the performance of the PIMS signature was within the 99^th^ percentile of the randomly generated signatures, thereby validating the robustness of PIMS selected signatures.

Whereas the experiments presented here focused on identifying prognostic and predictive gene signatures for breast cancer from microarray data, PIMS would also be appropriate to identify similar such signatures for different cancer types (lung, ovarian, colon…etc). Indeed, this notion is supported by our demonstration that PIMS can similarly identify prognostic gene signatures in colon cancer patients. Moreover, PIMS would readily function on other data formats as well, such as RNAseq data, or even copy number array data. Accordingly, we suggest that PIMS is broadly applicable to most commonly used high-throughput techniques used to profile tumors.

## Conclusions

We have built upon our previously published feature selection algorithm and packaged it into a freely accessible, user-friendly Excel file. Our data suggest that PIMS identifies gene signatures that are robustly associated with user defined clinical variables. Hence, PIMS represents a broadly applicable method to generate prognostic and predictive gene signatures that we expect will be highly useful to the research community.

## Electronic supplementary material

Additional file 1: Figure S1: The expression of each gene is standardized across the cohort of patients. The scoring of the gene is relative to the 95% confidence interval (A). Using the resulting expression value for each gene and the phenotype data we can calculate a predictive score for each gene. Table B illustrates how each combination affects the predictive score of each gene. Finally leave-one-out cross-validation (C) is used to find the best sized signature. (PDF 114 KB)

Additional file 2: **PIMS tutorial.** (DOCX 1 MB)

Additional file 3: **Pathway analysis of prognostic PIMS signature.** (XLSX 260 KB)

Additional file 4: Figure S2: PIMS identifies prognostic signatures in colon cancer (HR: 1.3, *p=0.0004, log-rank test) *test). (PDF 81 KB)
