# Supplementary material for: A program to identify prognostic and predictive gene signatures
Source: BMC Res Notes. 2014 Aug 18;7:546. doi: 10.1186/1756-0500-7-546 (PMC4148546; doi:10.1186/1756-0500-7-546)
Supplement: Supplementary file 1 — Additional file 1: Figure S1: The expression of each gene is standardized across the cohort of patients. The scoring of the gene is relative to the 95% confidence interval (A). Using the resulting expression value for each gene and the phenotype data we can calculate a predictive score for each gene. Table B illustrates how each combination affects the predictive score of each gene. Finally leave-one-out cross-validation (C) is used to find the best sized signature. (PDF 114 KB) [file 13104_2014_3077_MOESM1_ESM.pdf]

Chorlton, *et al*. Supplementary Figure 1

A

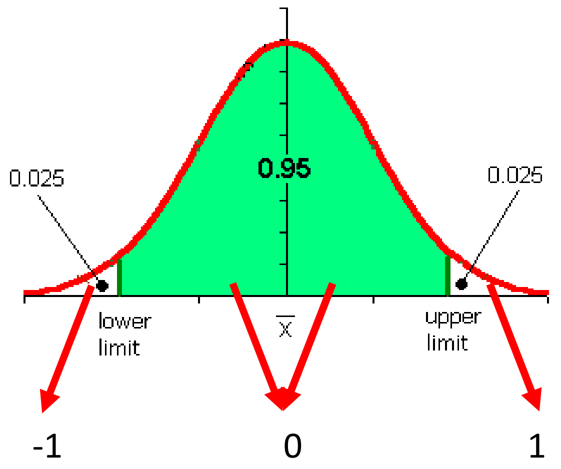

B

Gene expression of gene *x* \ Phenotype	**0 (Patient lived)**	**1 (Patient died)**
**-1**	Predictive score of gene *x* increased by 1	Predictive score of gene *x* decreased by 1
**0**	No change	No change
**1**	Predictive score of gene *x* decreased by 1	Predictive score increased by 1

C

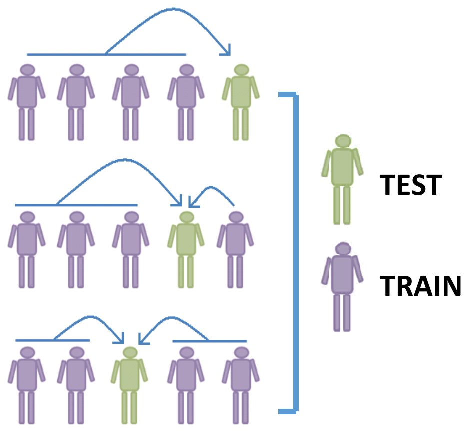

Supplementary figure 1. The expression of each gene is standardized across the cohort of patients. The scoring of the gene is relative to the 95% confidence interval (A). Using the resulting expression value for each gene and the phenotype data we can calculate a predictive score for each gene. Table B illustrates how each combination affects the predictive score of each gene. Finally leave-one-out cross-validation (C) is used to find the best sized signature.
