# Supplementary material for: A program to identify prognostic and predictive gene signatures
Source: BMC Res Notes. 2014 Aug 18;7:546. doi: 10.1186/1756-0500-7-546 (PMC4148546; doi:10.1186/1756-0500-7-546)
Supplement: Supplementary file 2 — Additional file 2: PIMS tutorial. (DOCX 1 MB) [file 13104_2014_3077_MOESM2_ESM.docx]

PIMS tutorial

Program start


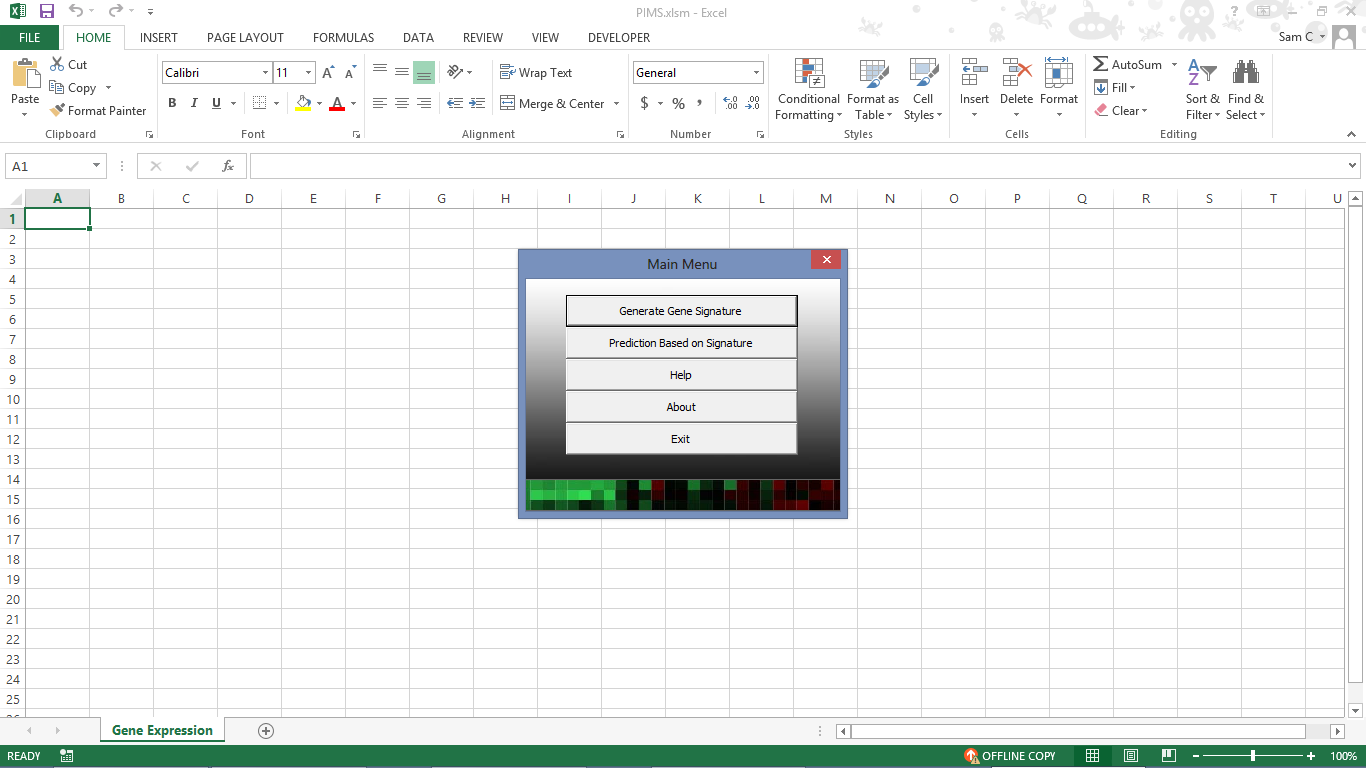


Click ‘Generate Gene Signature’


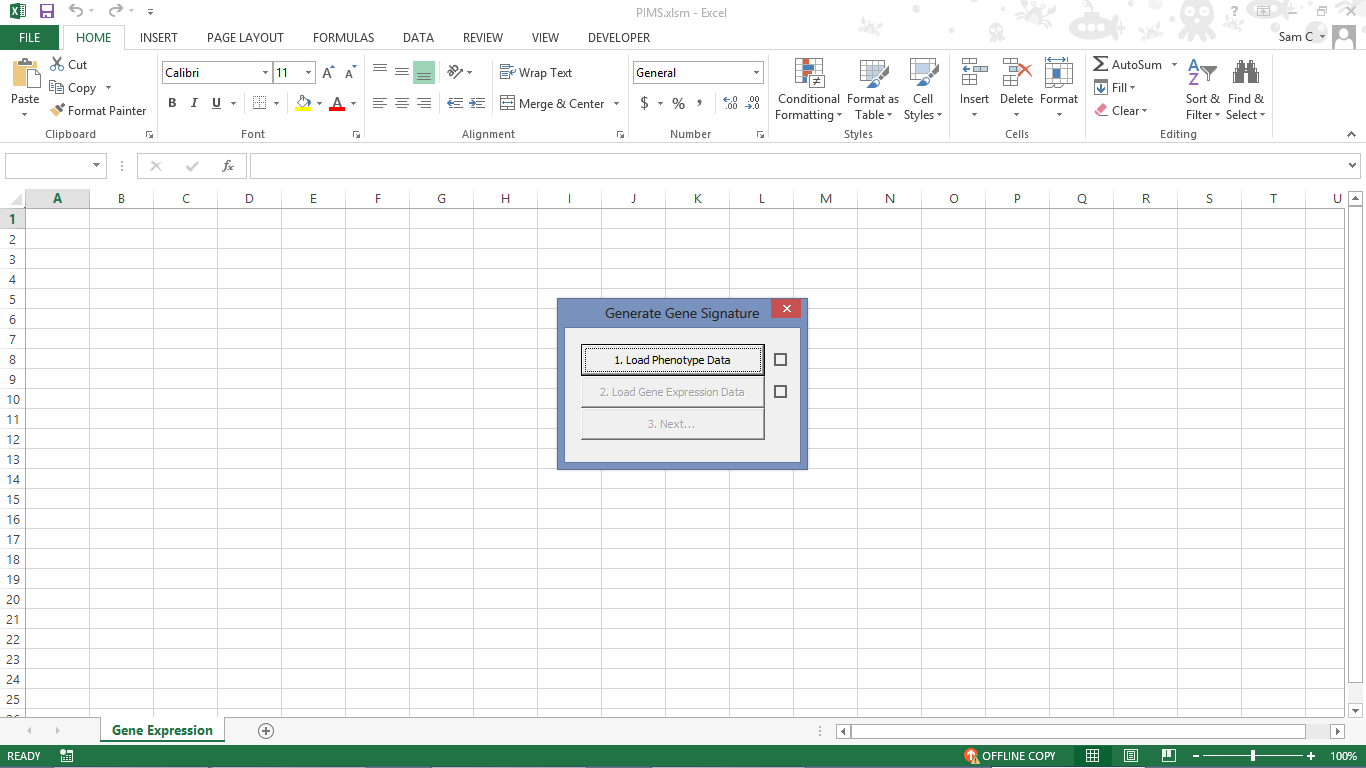


Click ‘Load Phenotype Dataset’


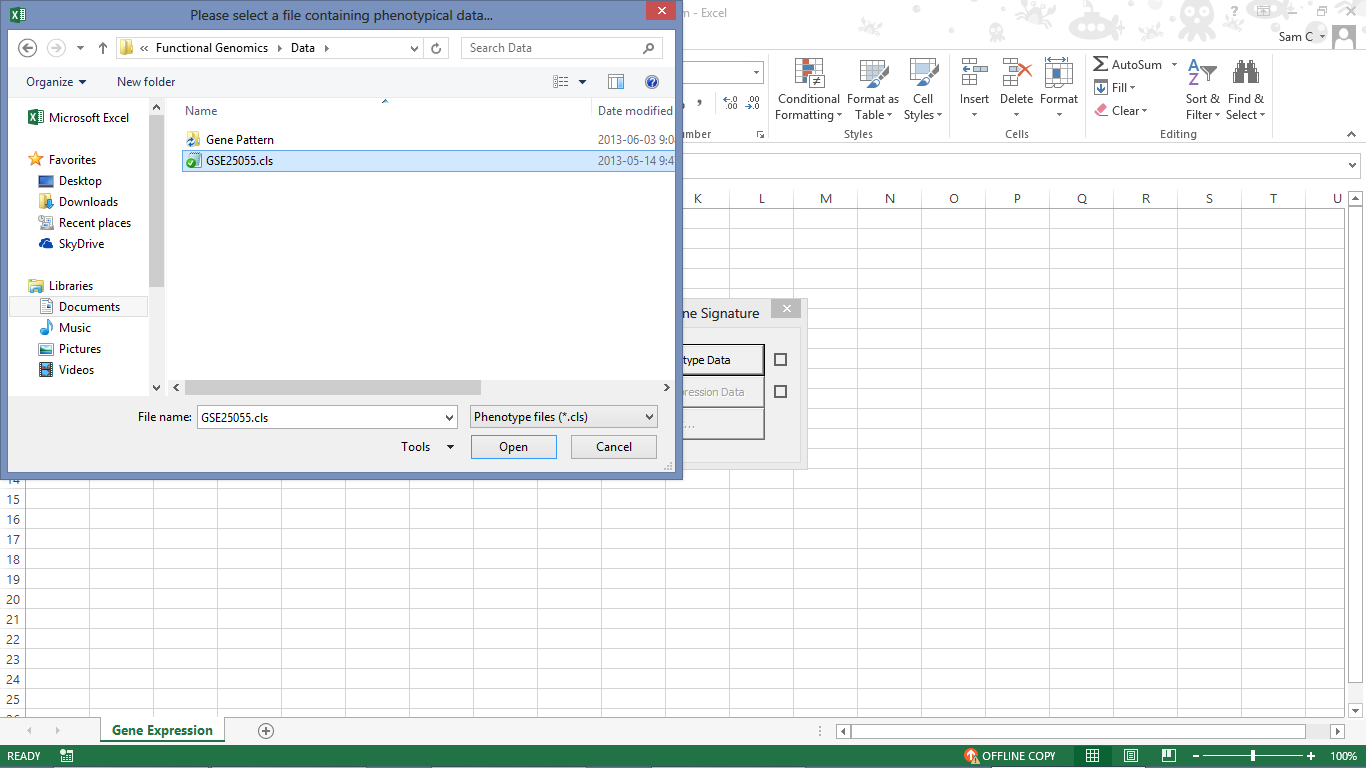


Select a .cls file. Note that the number of phenotypes and their order must match the gene expression file.

Click on ‘Load Gene Expression Dataset’.


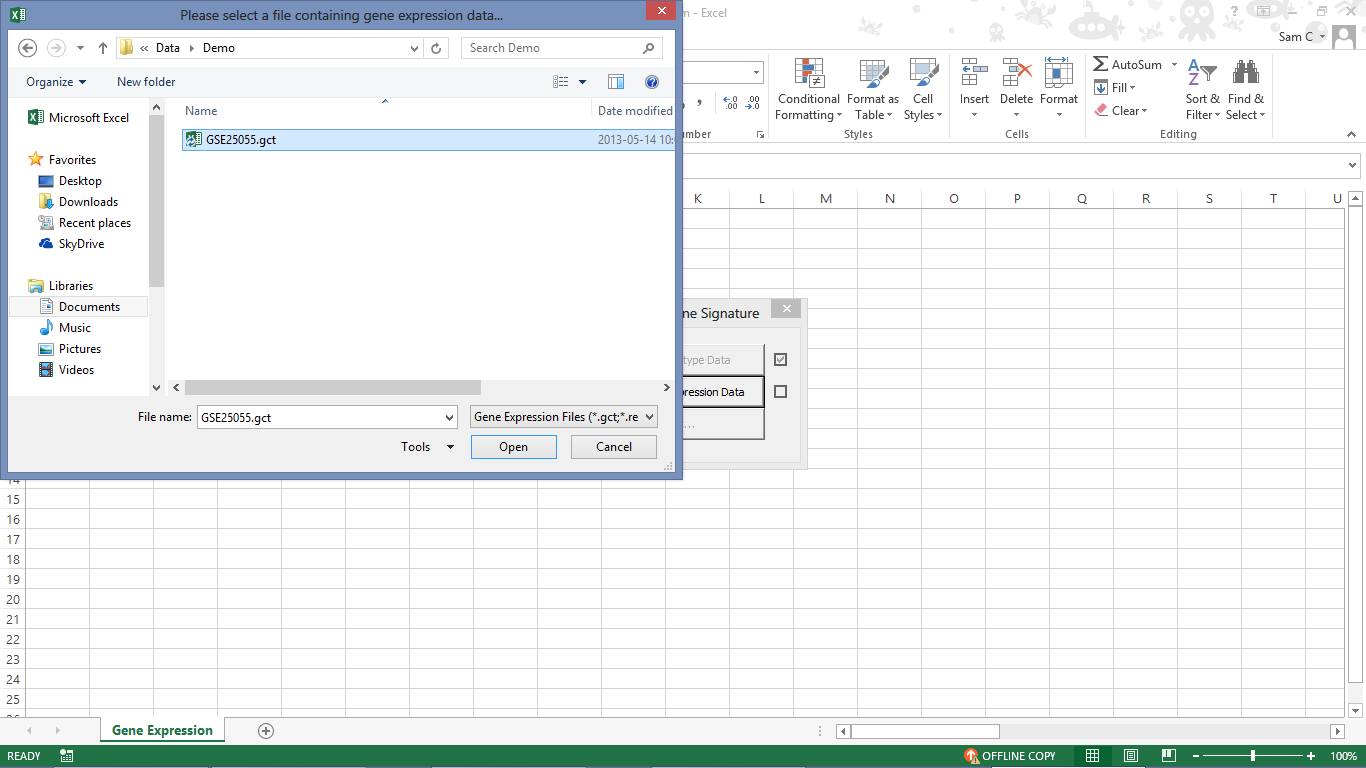


Load the gene expression file. Check the help section in the program to find the acceptable file formats.


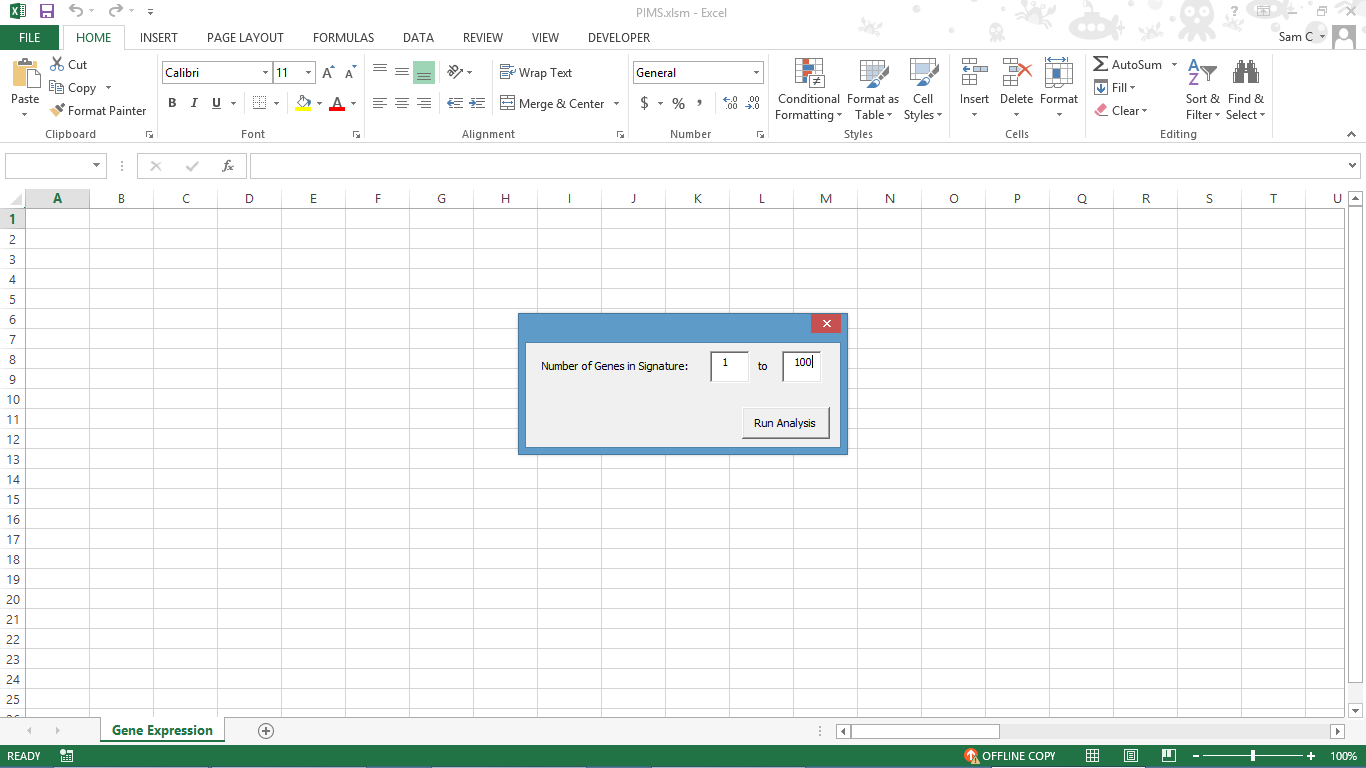


Enter in the requested size of the gene signature. Gene signatures with an even number of genes will be equally divided between the up regulated and down regulated genes. Gene signatures with an odd number of genes will have the extra gene added to the up regulated genes. Click on ‘Run Analysis’ to run the analysis.


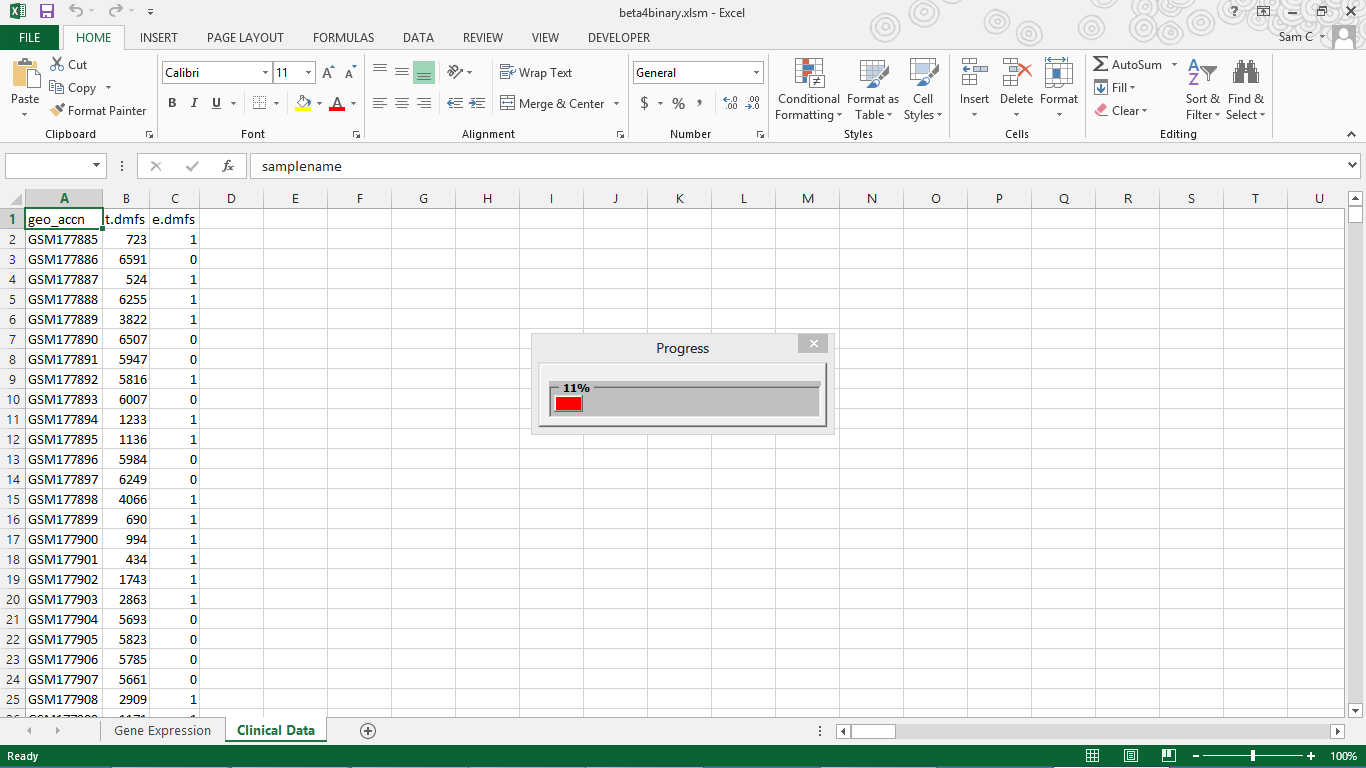


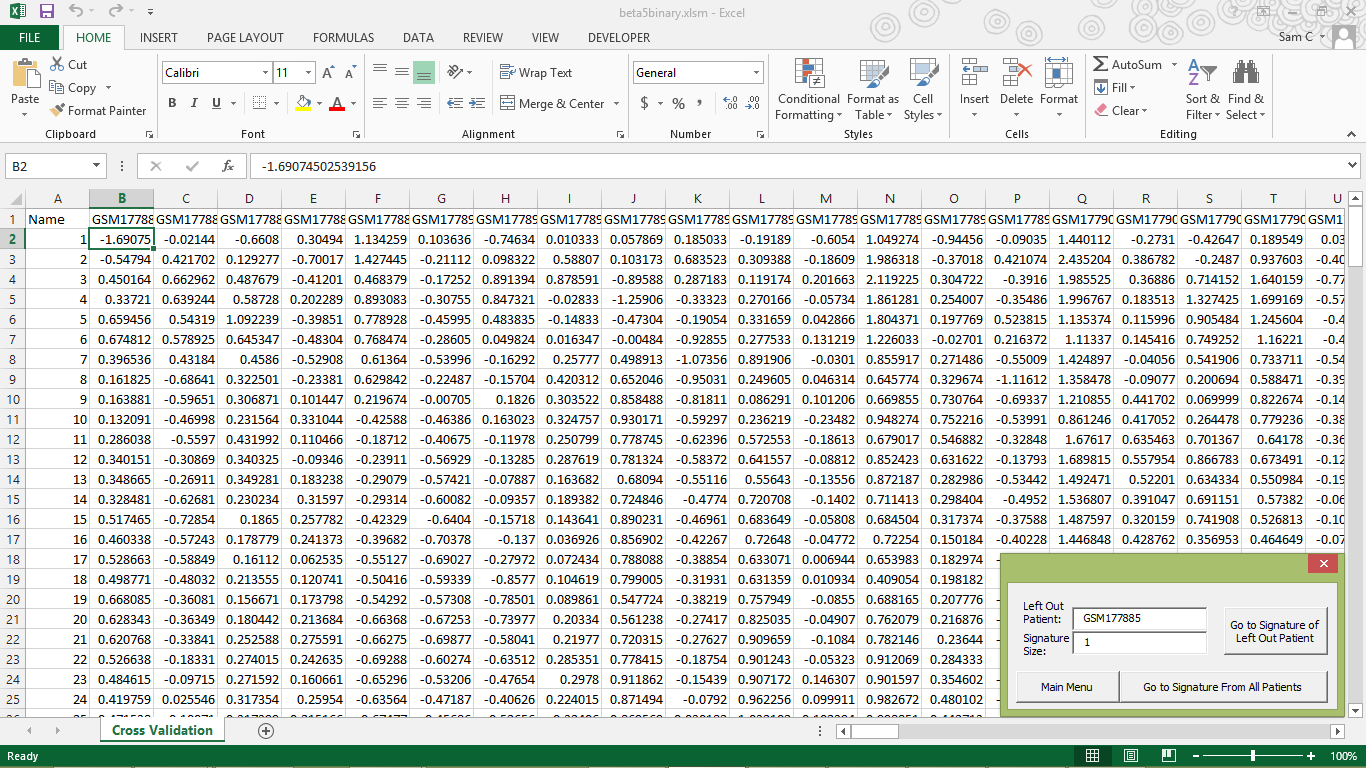


The results are outputted as leave-one-out cross-validated data. Row 1 contains the identifier of the patient that was left out, and column A contains the size of the gene signature. All other data is the predictive score of the left-out patient. Clicking on one of the scores will change the box in the right hand corner, enabling you to go to the signature that produced that score.

Sample Prediction Tutorial


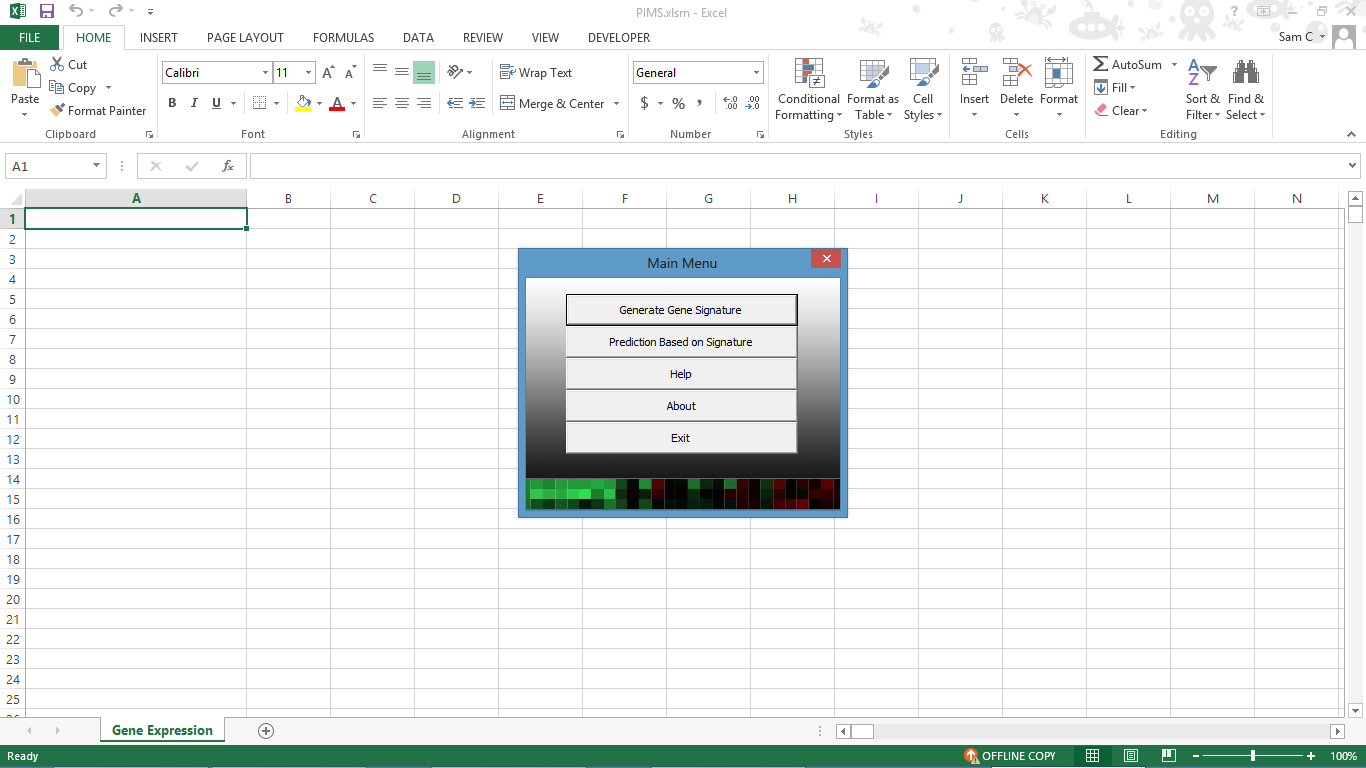


Click on ‘Prediction Based on Signature’.


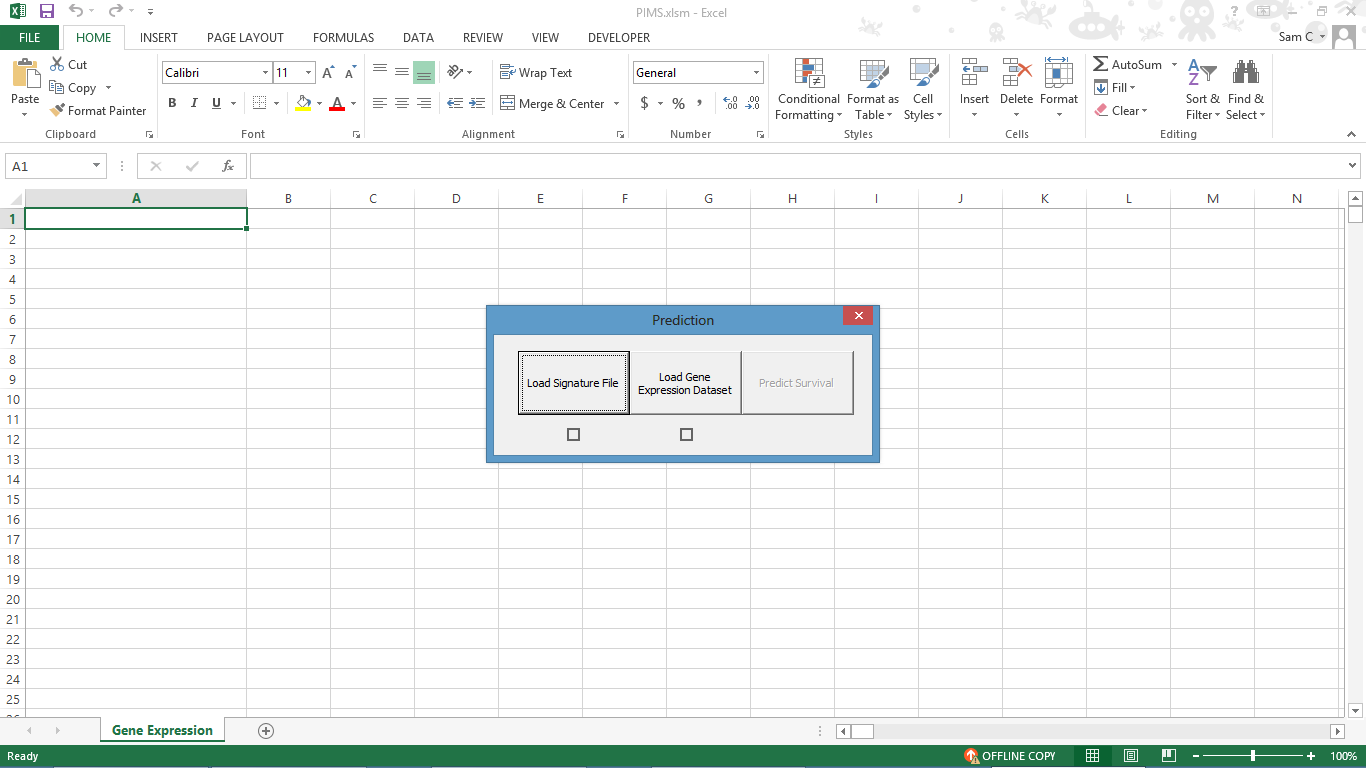


Click on ‘Load Signature File’. The signature file format is specified in the help sections of PIMS.


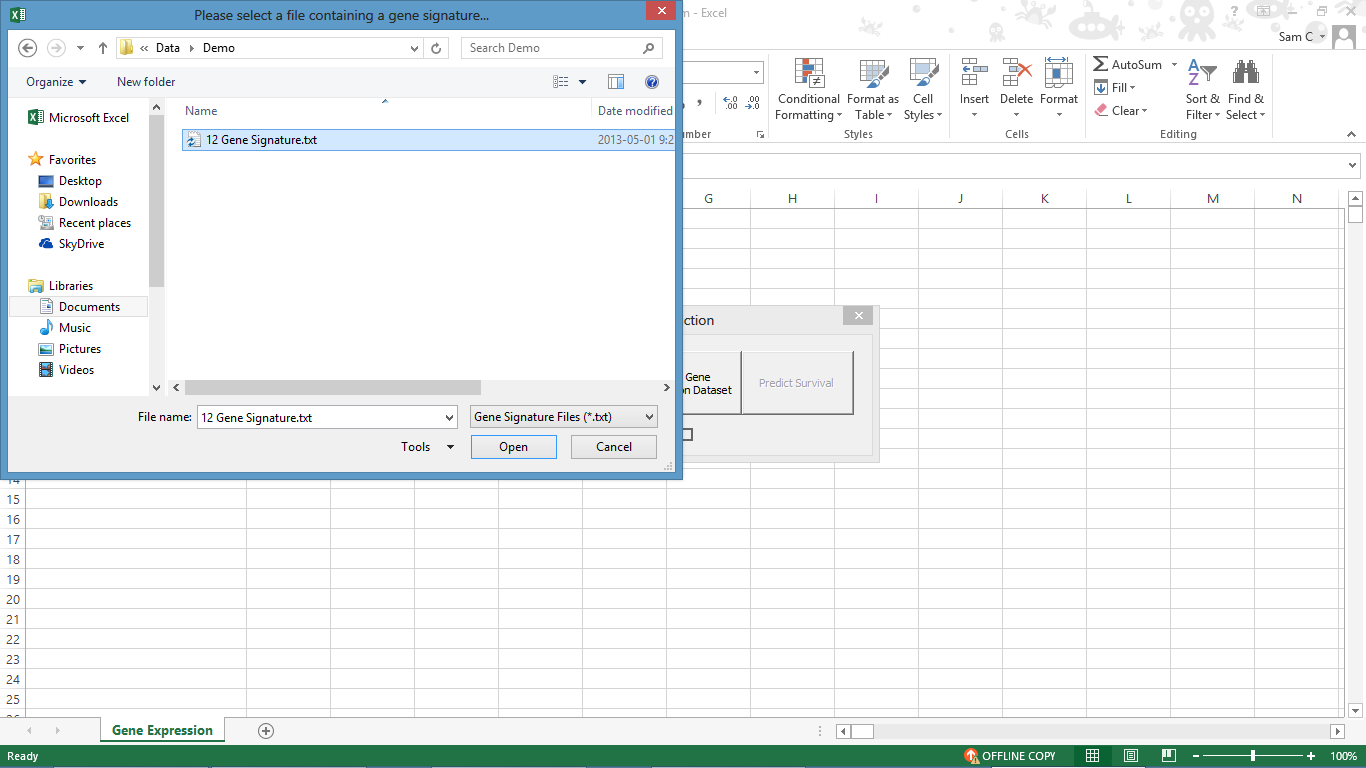


Load the signature.


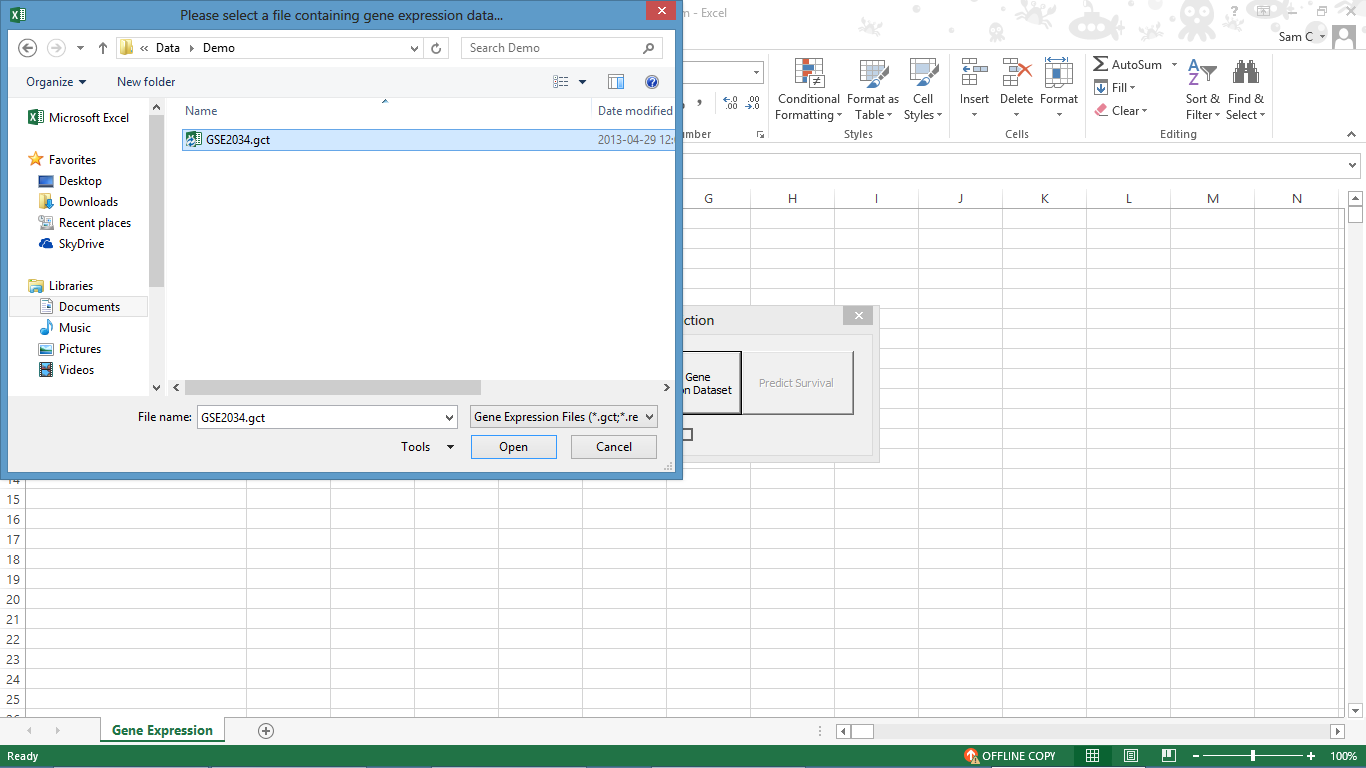


Click on ‘Load Gene Expression Dataset’ and load the file.


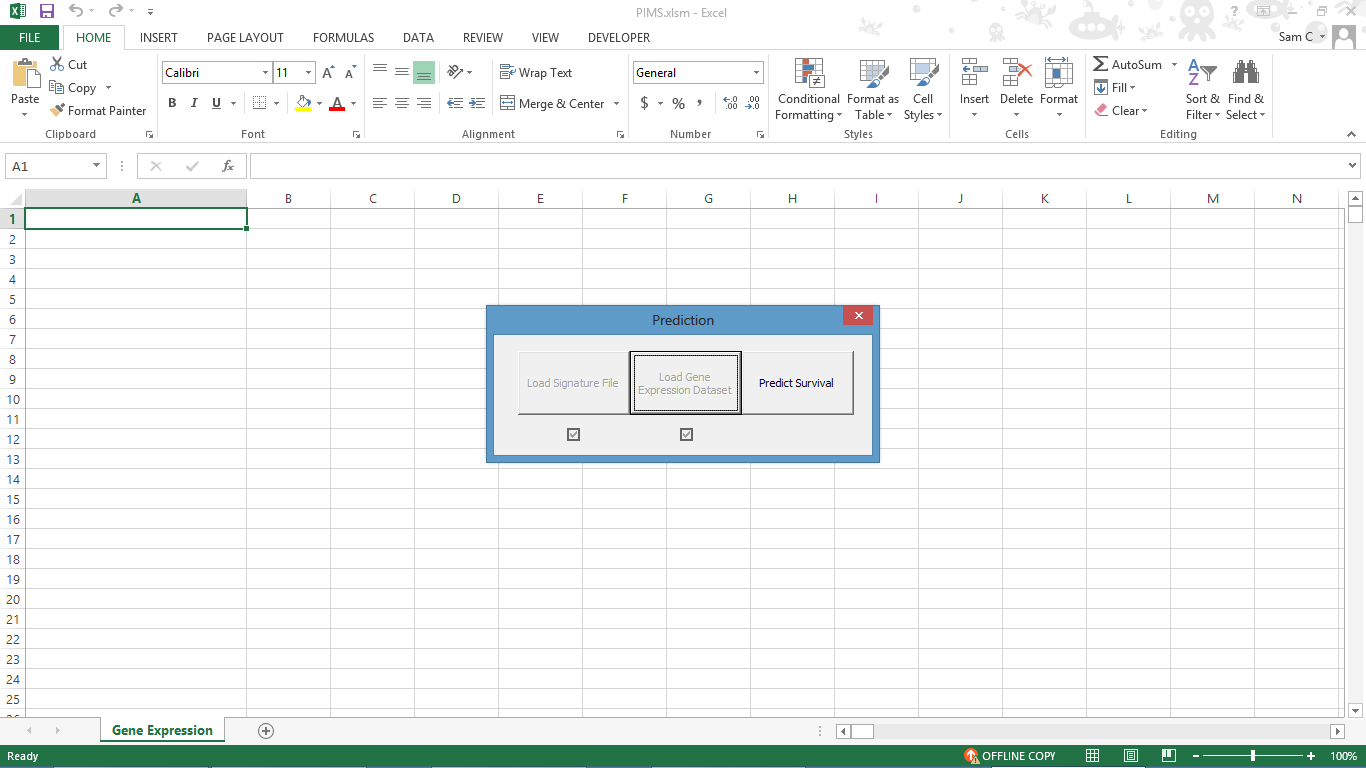


Click on ‘Predict Survival’.


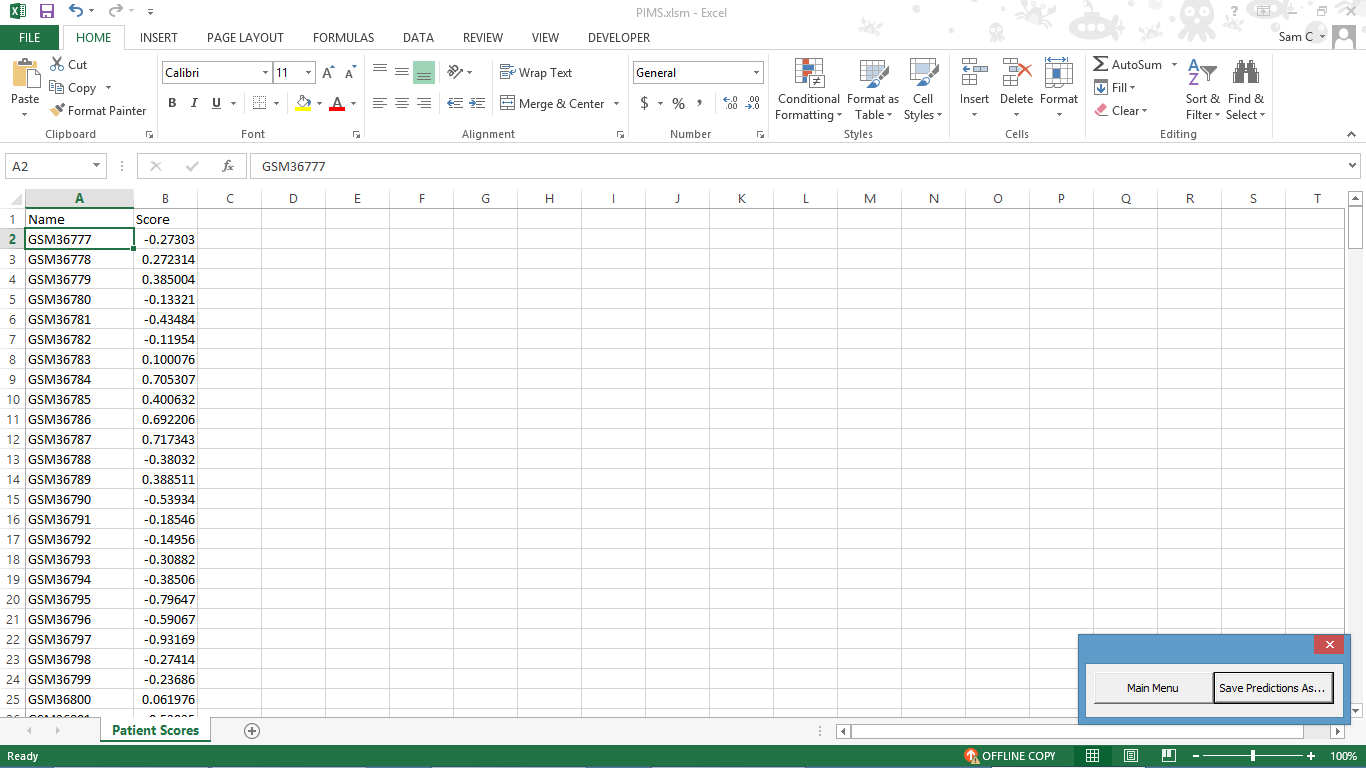


Sample/Patient Scores are outputted. See paper for more information on how scores are calculated.
