# Supplementary material for: A program to identify prognostic and predictive gene signatures
Source: BMC Res Notes. 2014 Aug 18;7:546. doi: 10.1186/1756-0500-7-546 (PMC4148546; doi:10.1186/1756-0500-7-546)
Supplement: Supplementary file 4 — Additional file 4: Figure S2: PIMS identifies prognostic signatures in colon cancer (HR: 1.3, *p=0.0004, log-rank test) *test). (PDF 81 KB) [file 13104_2014_3077_MOESM4_ESM.pdf]

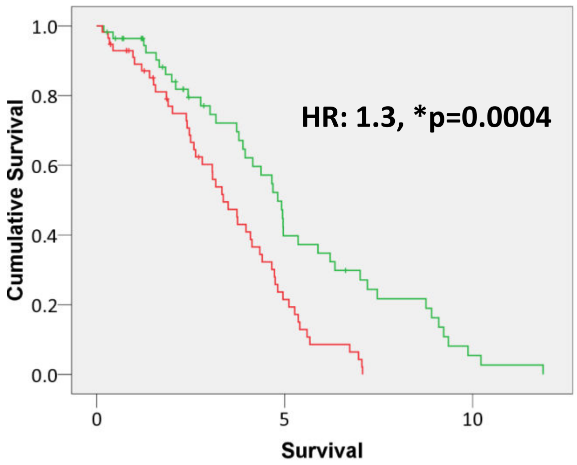

Supplementary figure 2. PIMS identifies prognostic signatures in colon cancer (HR: 1.3, *p=0.0004, log-rank test).
